# Роль контринсулярных гормонов в регуляции гомеостаза глюкозы и патогенезесахарного диабета 2-го типа при ХОБЛ

**DOI:** 10.14341/probl12566

**Published:** 2021-02-17

**Authors:** В. И. Кобылянский

**Affiliations:** Научно-исследовательский институт пульмонологии Федерального медико-биологического агентства

**Keywords:** ХОБЛ, контринсулярные гормоны, сахарный диабет 2-го типа

## Abstract

Частая сочетаемость сахарного диабета 2-го типа (СД2) и хронической обструктивной болезни легких (ХОБЛ) — социально значимая и далеко не изученная проблема. Однако ей посвящены лишь единичные работы. Для решения данной проблемы нами были проанализированы возможные патогенетические механизмы с позиции воздействия на гомеостаз глюкозы основных гормонов — инсулярных и контринсулярных. В настоящей работе внимание в этом плане больше сосредоточено на роли контринсулярных гормонов.Анализ проводился путем использования различных баз литературных данных, включая Index Medicus, Scopus, Pub Med, Embase, Кокрейна и другие, преимущественно за период 2000–2020 гг. Работы, посвященные непосредственно рассматриваемому аспекту, были опубликованы в основном в течение последних 5 лет.В результате анализа выявлено взаимоотягощающее влияние ХОБЛ и СД2 в случае сочетанного их протекания, в котором инициирующая роль принадлежит ХОБЛ. Также при этом выявлена значительная роль контринсулярных гормонов, во многом определяющая характер патогенеза СД2 при ХОБЛ. Кроме того, в статье обращается внимание на возможную роль генетических факторов, которые могут быть общими для ХОБЛ и СД2 и иметь немалое значение в коморбидности ХОБЛ и СД2. Полученные данные могут быть использованы как в диагностических, так и в терапевтических целях при коррекции нарушений углеводного обмена при ХОБЛ, что является уделом дальнейших исследований.

## Abstract (en)

The frequent combination of type 2 diabetes mellitus (T2DM) and chronic obstructive pulmonary disease (COPD) is an important socially significant and far from being studied problem. However, only a few works are devoted to it. To solve this problem, we analyzed the possible pathogenetic mechanisms from the standpoint of the impact on glucose homeostasis of the main hormones — insular and contrainsular.The analysis was carried out using various literature databases, including Index Medicus, Scopus, Pub Med, Embase, Cochrane and others for the period, with rare exceptions, for 2000–2020, of which the works devoted directly to the aspect considered in this work were published in the last 5 years.The analysis revealed a mutual aggravating effect of COPD and T2DM, in which COPD plays an initiating role. It also revealed a significant role of counterinsular hormones, which largely determines the nature of the pathogenesis of T2DM in COPD.In addition, the article draws attention to the possible role of genetic factors that can be common for COPD and T2DM and have a significant role in the comorbidity of COPD and T2DM. The data obtained can be used for both diagnostic and therapeutic purposes in the correction of disorders of carbohydrate metabolism in COPD, which is the lot of further research.

Если рассматривать гормональную систему регуляции гомеостаза при хронической обструктивной болезни легких (ХОБЛ) как состоящую из двух групп гормонов — инсулярных и контринсулярных, то можно отметить, что в этом плане исследования посвящены главным образом проблеме состояния инсулярного аппарата поджелудочной железы [1–4]. В настоящей работе внимание будет акцентировано в основном на роли контринсулярных гормонов (КИГ) в гомеостазе глюкозы и в патогенезе сахарного диабета 2 типа (СД2) при ХОБЛ. Роль КИГ с позиции пульмонологии исследована фрагментарно и отражена лишь в единичных работах, а имеющиеся данные противоречивы. При этом предполагается, что они играют далеко не последнюю роль, если учесть то, что гиперпродукция КИГ, как это наблюдается при ряде эндокринопатий, способствует повышению сахара в крови и развитию сахарного диабета [5–9]. Выраженная гипергликемия, особенно в условиях нарушения так называемой «пластичности» β-клеток (способности их массы адаптироваться к потребностям организма в инсулине), неблагоприятно воздействует на инсулярный аппарат, еще больше подавляя выработку инсулина [10]. Таким образом формируется своеобразный «порочный круг», требующий уточнения.

## РОЛЬ КОНТРИНСУЛЯРНЫХ ГОРМОНОВ В РЕГУЛЯЦИИ ГОМЕОСТАЗА ГЛЮКОЗЫ

Действие КИГ противоположно таковому инсулина. Их выработка является защитной реакцией на низкий уровень глюкозы в крови, что позволяет поддерживать его в нормальных пределах. Наряду с гормонами с эффектом явной контринсулярной направленности существуют гормоны, которые при определенных условиях обладают подобным эффектом, в частности андрогены, к которым относят тестостерон. КИГ всего несколько, и их основные биологические эффекты, наряду с упомянутыми андрогенами, представлены в таблице.

**Table:** Таблица 1. Ведущие биологические эффекты некоторых из основных контринсулярных и других гормонов подобного действия

Гормоны	Продуцирование и механизмы действия
Соматотропный гормон (гормон роста)	Вырабатывается гипофизом. Активирует глюконеогенез, гликогенолиз, кетогенез в печени, а также липолиз жировой ткани (через повышение чувствительности к адреналину и тиреоидным гормонам). Увеличивает инсулинорезистентность (за счет активации инсулиназы печени) и контринсулярное действие глюкокортикоидов
Гормоны щитовидной железы (тироксин, трийодтиронин)	Активируют гликогенолиз в печени и мышцах и действие гексокиназы в кишечнике, обусловливая усиление всасывания глюкозы в кровь. Усиливают липолиз и тормозят образование и отложение жира. Увеличивает инсулинорезистентность (за счет активации инсулиназы печени)
Глюкагон	Вырабатывается альфа-клетками островков Лангерганса поджелудочной железы. Увеличивает глюконеогенез, гликогенолиз, кетогенез в печени и липолиз в жировой ткани. Тормозит поглощение глюкозы тканями
Глюкокортикоиды (кортизол и др.)	Вырабатываются в корковом слое надпочечников. Активируют в печени глюконеогенез и тормозят потребление глюкозы периферическими тканями, блокируя действие инсулина на уровне мембранных ферментов-переносчиков. Увеличивают липолитический эффект адреналина и соматотропного гормона в жировой ткани
Адреналин	Вырабатывается в мозговом слое надпочечников. Активирует гликогенолиз в печени и мышцах и тормозит поглощение глюкозы тканями. Увеличивает липолиз жировой ткани и секрецию глюкагона
Андрогены* (тестостерон)	Вырабатываются в корковом слое надпочечников, а также в семенниках. Низкий уровень тестостерона увеличивает инсулинорезистентность. У женщин гиперандрогенные состояния (синдром поликистоза яичников) увеличивают инсулинорезистентность и риск СД2

При действии КИГ происходит активизация ферментов, способствующих снижению функциональной активности инсулина и его содержания, а также увеличению процессов инсулинорезистентности тканей и органов-мишеней. Избыточное образование КИГ обеспечивает торможение гликогенеза, активацию гликогенолиза, липолиза в жировых депо, а увеличение количества таких КИГ, как глюкокортикоиды и тироксин, вызывает активацию протеолиза в тканях и глюконеогенеза в печени [11].

На экспериментальных моделях, а затем и клинически была показана значимость КИГ в развитии нарушений углеводного обмена, механизмы которых могут быть причастны и к СД2 [12]. Все эти гормоны вначале усиливают секрецию инсулина, но при длительном введении приводят к истощению инсулярного аппарата и деструктивным изменениям в базофильных инсулоцитах. Каждый из этих гормонов обладает своеобразным действием на углеводный обмен. Но в качестве яркого примера, свидетельствующего о значительной роли КИГ в развитии СД2, чуть подробнее коснемся развития гипергликемии натощак, обусловленной поражением гипоталамуса и/или гипофиза с гиперсекрецией соматотропного (СТГ) и адренокортикотропного гормонов. При этом имеют место гипофизарные опухоли или увеличение секреции соматолиберина [13]. Углеводный обмен при гиперсекреции СТГ характеризуется усилением секреции глюкагона и инсулина. Параллельно со стимуляцией печеночной инсулиназы и гликогенолиза ограничивается периферическое использование глюкозы, наступают гипергликемия с глюкозурией. Соматотропин обладает пермиссивным действием по отношению к инсулину и глюкокортикоидам. Поэтому малые дозы СТГ способствуют анаболизму, а большие — катаболизму. В итоге при гиперсекреции СТГ нарушения углеводного обмена представляют собой картину инсулинорезистентного диабета. Гиперсекреция гормона роста приводит к усилению процессов глюконеогенеза и липолиза, сопровождающихся снижением проницаемости клеточных мембран для глюкозы, усилением секреции инсулина с постепенным истощением инсулярного аппарата. При этом имеют место симптомы гиперфункции гипофиза (акромегалия, гигантизм), сухость слизистых оболочек и кожи, жажда, полиурия [13]. В крови повышено содержание гормона роста, выявляются гипергликемия, гликозурия, повышение содержания свободных жирных кислот. Основными принципами терапии являются лечение основного заболевания, а также диетотерапия с ограничением легкоусвояемых углеводов, иногда возможна инсулинотерапия. Таким образом, инсулиновая недостаточность, обусловленная особенностями СТГ, может лежать в основе развития гипергликемии и СД2. При этом инсулиновая недостаточность может быть также следствием самой гипергликемии, так как продукты перекисного окисления липидов, имеющие место при данной патологии, способны приводить к дистрофии и гибели бета-клеток поджелудочной железы, являющейся высокочувствительной к оксидативному стрессу [14].

Инсулинорезистентность (ИР), развивающаяся в результате вышеуказанных факторов и являющаяся ведущим патогенетическим механизмом нарушений углеводного обмена при ХОБЛ, и неадекватная секреция инсулина могут усиливать друг друга. В результате компенсаторной гиперинсулинемии возрастают ИР и потребность в выработке инсулина, что ведет к развитию гипергликемии. Гипергликемия приводит к окислительному стрессу, вызванному аутоокислением глюкозы, и вызывает повреждения фосфолипидного слоя плазматических мембран бета-клеток и тканей-мишеней. Это способствует прогрессированию ИР, секреторные возможности инсулярного аппарата снижаются из-за апоптоза бета-клеток [15]. Таким образом, создается многоуровневый патологический круг, на фоне которого имеет место коморбидность. Как показали проведенные исследования, окислительный стресс у пациентов с риском развития нарушений углеводного обмена может обусловливать ИР еще за 10–15 лет до клинических проявлений нарушения гликемии [16].

## ПАТОГЕНЕТИЧЕСКИЕ МЕХАНИЗМЫ КОМОРБИДНОСТИ ХРОНИЧЕСКОЙ ОБСТРУКТИВНОЙ БОЛЕЗНИ ЛЕГКИХ И САХАРНОГО ДИАБЕТА 2 ТИПА

Было показано, что ХОБЛ является фактором риска СД2. Согласно современным представлениям, ХОБЛ является системным заболеванием, вовлекающим в патологический процесс многие органы и системы. В его основе лежат такие факторы, как хронический воспалительный процесс, не только касающийся респираторного тракта, но и имеющий системный характер, а также гипоксемия, курение, малоподвижный образ жизни и др. Однако они являются и причиной развития СД2. Именно эта общность в патогенезе указанных заболеваний и характер причинно-следственной связи и легли в основу нашей гипотезы о том, что ХОБЛ является фактором риска СД2. При этом данный фактор встречался с высокой достоверностью чаще у больных ХОБЛ (р<0,001), чем в популяции, тогда как в когорте больных СД2 показатели распространенности ХОБЛ не превышали контрольных значений [17]. На данном этапе исследования ограничивались представлением о том, что ведущая роль в инициации механизмов, приводящих к развитию СД2, принадлежит ИР [1]. Исследования другой обструктивной патологии легких, обусловленной аллергическим воспалением при бронхиальной астме, исключали подобную связь [2].

Целый ряд работ подтверждает гипотезу о наличии тесной взаимосвязи ХОБЛ и СД2 и их патогенетических механизмов. Связь между этими двумя болезнями сложна, многофакторна и не всегда понятна, но ее изучение и раскрытие могут оказать положительное влияние на подход к лечению этих высокозначимых социальных заболеваний. Понимание сути их частого совместного протекания важно для совершенствования и разработки новых методов диагностики и терапевтических подходов к этим заболеваниям [18]. В частности, СД2 может негативно влиять на качество жизни и исходы ХОБЛ, учитывая наличие потенциальных общих связей между ними, таких как системное воспаление, окислительный стресс, гипоксемия и гипергликемия [19]. На выраженную негативную роль коморбидности ХОБЛ и СД2 указывают исследования Ho T.W. и соавт. (2017) [20], которые провели когортное исследование пациентов из базы данных долгосрочного медицинского страхования на Тайване в период с 2000 по 2013 гг. Для идентификации факторов риска, ассоциированных с СД2, и оценки прогноза у пациентов с СД2 и ХОБЛ применялась модель пропорциональных рисков Кокса. Диагностированный ранее СД2 имел место у 332 (16%) пациентов с ХОБЛ, у которых выявлялся значительно более высокий коэффициент риска [ 1,244, 95% доверительный интервал (ДИ) 1,010–1,532] в отношении смертности, чем у пациентов только с ХОБЛ. В течение 10-летнего периода наблюдения у 304 (19%) из 1568 пациентов с ХОБЛ развился СД. Выживаемость у пациентов с ХОБЛ и СД была меньшей, чем в контрольной группе (p=0,027) [20].

СД2, по различным оценкам, поражает 2–37% пациентов с ХОБЛ. Компенсация СД2 также может коррелировать с качеством жизни и функцией внешнего дыхания (ФВД). Для изучения этой корреляции у больных, поступивших в стационар с обострением ХОБЛ, Mekov E.V. и соавт. (2016) [21] провели исследование, в которое были включены 152 пациента. Всем была выполнена спирометрия. 13,2% (20/152) пациентов имели давний стаж СД2, у 21,7% (33/152) СД2 был диагностирован впервые, а у 30,9% (47/152) имел место преддиабет. Нелеченый СД2 ассоциировался как со снижением качества жизни, так и c ухудшением ФВД. Уровень гликозилированного гемоглобина (HbA1c) отрицательно коррелировал с ФВД, а пациенты, госпитализированные с обострением ХОБЛ, имели высокий риск нарушения углеводного обмена [21]. Gläser S. и соавт. (2015) [22] провели поиск литературы, посвященной взаимосвязи ХОБЛ и СД2, используя базы данных PubMed. Полученные результаты свидетельствуют о значительном взаимоотягощающем влиянии этих заболеваний. При этом СД2 может способствовать прогрессированию и ухудшению прогноза ХОБЛ, что может быть результатом прямого воздействия на легкие механизмов воспаления или восприимчивости к бактериальной инфекции и гипергликемии. И наоборот, ХОБЛ увеличивает риск развития СД2 вследствие воспалительных процессов и/или побочных эффектов, связанных с применением высоких доз кортикостероидов [22].

Для оценки распространенности сопутствующих заболеваний у пациентов был проведен метаанализ 11 исследований, включавших 47 695 183 пациента с ХОБЛ и 47 924  876 пациентов без ХОБЛ. Средний возраст пациентов с ХОБЛ составил 66,66±8,72 года, 55,4±11,9% пациентов составляли мужчины. Распространенность СД2 (отношение шансов (ОШ) 1,22; 95% ДИ 1,07–1,38; р=0,003) оказалась значительно выше у пациентов с ХОБЛ, чем в контрольной группе [23]. Оценивая распространенность СД2 при ХОБЛ, Mannino D.M. и соавт. (2008) [24] проанализировали данные 20 296 пациентов в возрасте ≥45 лет. Выборка была стратифицирована на основе данных о ФВД в соответствии с критериями Глобальной инициативы по диагностике, лечению и профилактике ХОБЛ. В моделях логистической регрессии, учитывающих возраст, пол, расу, курение, индекс массы тела и образование, у субъектов с 3-й или 4-й стадией ХОБЛ имела место более высокая распространенность СД2 (ОШ 1,5; ДИ 95% 1,1–1,9). Эти данные ассоциировались с высокой частотой госпитализации и смертности, которая была выше у людей с нарушениями ФВД. Нарушение ФВД также связывалось с более высоким риском развития коморбидной патологии [24].

С целью изучения связи ХОБЛ с риском развития СД2 Rasmussen S.M. и соавт. (2018) [25] были проведены систематический обзор и метаанализ когортных и тематических исследований, которые осуществлялись двумя авторами независимо друг от друга. Скорректированные данные объединялись с использованием модели со случайными эффектами для расчета суммарных коэффициентов шансов и соответствующих 95% ДИ. Было выделено четыре когортных и три исследования «случай-контроль», в которых приняли участие 1 369 560 пациентов, из которых 42 716 страдали ХОБЛ. Метаанализ скорректированных данных из всех семи исследований показал, что в группе ХОБЛ риск развития СД2 оказался выше, чем в группе без нее [25].

Легочная гипертензия (ЛГ) является распространенным осложнением ХОБЛ. Недавние исследования показали, что СД2 является плохим прогностическим фактором у пациентов с ХОБЛ, однако связь между СД2 и ЛГ при хронических респираторных заболеваниях остается неясной. Takahashi T. и соавт. (2018) [26] попытались выяснить, является ли СД2 предиктором ЛГ у пациентов с ХОБЛ. В проспективном анализе приняли участие 386 пациентов. Показатели градиента давления трикуспидальной регургитации при трансторакальной эхокардиографии, свидетельствующие о ЛГ, составили ≥40 мм рт. ст. Сравнивались клинические характеристики и влияние СД2 на пациентов с ХОБЛ и без нее. Из 386 пациентов у 42 (10,9%) была диагностирована ЛГ; у них же отмечалась более высокая степень частоты осложнений СД2. Многовариантный логистический регрессионный анализ показал, что наличие СД (ОШ 2,935; 95% ДИ -1,505–5,725; р=0,002) ухудшало ЛГ у больных с ХОБЛ. Таким образом, СД2 не только ассоциировался с ЛГ, но и явился независимым фактором прогнозирования ЛГ у пациентов с ХОБЛ [26]. Lee C.T. и соавт. (2013) попытались выяснить, является ли ХОБЛ фактором риска СД2 в азиатской популяции [27]. Из национальной базы данных исследований в области медицинского страхования Тайваня были проанализированы данные 16 088 пациентов, включая 8044 пациента с ХОБЛ и 8044 — без нее. За время 5,5-летнего наблюдения у пациентов с ХОБЛ был выявлен значительно более высокий уровень заболеваемости СД2, чем в контрольной группе (р<0,001). ХОБЛ была достоверно ассоциирована с риском развития СД2 (ОШ 1,41; ДИ 1,23–1,63; р<0,001) после корректировки на пол, возраст, социальные условия, страховые взносы, лечение стероидами, наличие гипертонии, ИБС и цереброваскулярных заболеваний [27].

Кроме мнения, подтверждающего роль хронического воспаления, гипоксии и других факторов, присущих как для ХОБЛ, так и для СД2, выдвинуто предположение, что связь между СД2 и ХОБЛ в немалой степени может быть объяснена наличием общих генетических факторов. Это соотносится с информацией о том, что ХОБЛ — одна из главных причин заболеваемости и смертности в мире и связана с некоторыми системными заболеваниями, например, СД2. В 2015 г. Meteran H. и соавт. провели исследование, целью которого стало изучить связи между СД2 и ХОБЛ у близнецов во взрослом возрасте и определить, насколько сопутствующая патология между этими заболеваниями связана с общими факторами генетики или экологии [28]. Были сопоставлены данные вопросника по хроническому бронхиту (ХБ) у 13 649 близнецов в возрасте 50–71 года из Датского реестра близнецов и данные о СД2 из Датского национального реестра пациентов. У лиц с ХБ риск развития СД2 оказался больше, чем без него (3,5 против 2,3%), OШ=1,57; ДИ 1,10–2,26; р=0,014. Еще более высоким он оказался при ХОБЛ по сравнению с пациентами без нее (6,6 против 2,3%), OШ=2,62; ДИ 1,63–4,2; р<0,001 [28]. Результаты остались прежними после поправки на возраст, курение, пол и ИМТ. При этом, согласно классическому моделированию с помощью близнецов, предполагалось, что на данный фенотипический признак влияют генетические факторы и факторы окружающей среды. Эти факторы могут быть разложены на аддитивные генетические воздействия (А), представляющие собой сумму эффектов всех аллелей, влияющих на признак, негенетические влияния (D), представляющие взаимодействия между аллелями на одном и том же или разных локусах, общие факторы окружающей среды (C) и уникальные экологические факторы (E). Была применена специальная генетическая модель (АСЕ) для анализа, позволяющая оценить генетическую и экологическую взаимосвязь между ХБ, ХОБЛ и СД2. Исследование показало, что у лиц с ХБ и в большей степени с ХОБЛ повышен риск развития СД2 в 1,5 и 2,5 раза соответственно. Было показано, что С-реактивный белок, являющийся общим неспецифическим маркером воспаления и наличия сопутствующей патологии (в том числе кардиоваскулярной), значительно подвержен влиянию генетических детерминант [29]. При этом последние объясняют 33% и 43% соответственно корреляции между ХБ и СД2 и между ХОБЛ и СД2. Однако результаты не были статистически значимыми, вероятно, из-за небольшого числа пораженных пар близнецов, особенно с СД2 [28].

Отраженный выше анализ свидетельствует о несомненном взаимоотягощающем влиянии ХОБЛ и СД2. При этом с позиции первичности, учитывая данные, полученные нами ранее, мы все же больше склоняемся к мнению о том, что имеет место не случайное сочетание протекания во времени СД2 и ХОБЛ, а коморбидность, инициатором, причинным фактором в которой выступает ХОБЛ. И это не противоречит пониманию, отраженному выше.

Однако надо отметить, что хотя СД2 является распространенной сопутствующей патологией у пациентов с ХОБЛ, все еще остаются вопросы относительно связи между этими заболеваниями. Rogliani P. и соавт. (2014) ретроспективно оценили данные пациентов, страдающих ХОБЛ, в период между 2010 и 2012 гг. [30]. Исследуемая популяция была проанализирована по группам с учетом возраста, пола, индекса массы тела (ИМТ), статуса курения, функции легких, сопутствующих заболеваний и получаемой фармакотерапии. Из 493 пациентов с ХОБЛ 92 (18,7%) страдали СД2 без существенных гендерных различий. Распространенность была одинаковой среди разных возрастных групп, но связь была сильнее у пациентов моложе 65 лет. Значимая ассоциация имела место только у пациентов с ожирением и у пациентов с ХОБЛ средней и тяжелой степени, но не с легкой степенью ХОБЛ. Ассоциация с СД2 была более выраженной у пациентов с ХОБЛ по сравнению с общей популяцией и коррелировала с увеличением ИМТ и наличием других сопутствующих заболеваний, что позволяет предположить, что оба заболевания могут быть мишенью системного воспаления [30].

## РОЛЬ КОНТРИНСУЛЯРНЫХ ГОРМОНОВ В НАРУШЕНИИ ГОМЕОСТАЗА ГЛЮКОЗЫ И ИХ ИЗУЧЕНИЕ ПРИ ХРОНИЧЕСКОЙ ОБСТРУКТИВНОЙ БОЛЕЗНИ ЛЕГКИХ

Роль КИГ в гомеостазе глюкозы и развитии гипергликемии информативно прослеживается на фоне синдрома гиперметаболизма при критических состояниях у лиц без диабета, когда имеет место повышение уровня КИГ [31–33]. Было показано, что в механизмы, лежащие в основе развития гипергликемии, входит выброс катехоламинов, кортикостероидов и медиаторов, участвующих в процессах воспаления. У таких больных гипергликемия может быть вызвана и ятрогенными причинами (например, введением симпатомиметиков или глюкокортикоидов) [33]. С повышением уровня КИГ происходит активация липолиза, протеолиза и цикла Кори. Изменения пострецепторного сигнала в клетках скелетной мускулатуры происходят из-за ингибиции пируватдегидрогеназы. Это ключевой фермент, посредник между циклом трикарбоновых кислот и путем гликолиза Эмбдена–Мейергофа. Снижение активности пируватдегидрогеназы приводит к неполному окислению глюкозы, способствует накоплению пирувата и стимуляции глюконеогенеза, что, в свою очередь, вызывает развитие гипергликемии [34]. Таким образом, создается порочный круг: длительное системное воспаление при ХОБЛ сопровождается оксидативным стрессом, который способствует развитию ИР, гипергликемии. На фоне гипергликемии повреждаются бета-клетки, что, прогрессируя, способствует развитию СД2 или усугубляет течение патологического процесса при его наличии.

Среди КИГ определенную значимость представляют и гормоны щитовидной железы и коры надпочечников. Это необходимые и взаимосвязанные общеадаптационные элементы организма, чем и вызвана целесообразность их одновременного изучения. Существует не очень много данных о гормонах коры надпочечников в крови при ХОБЛ, и они весьма противоречивы.

Кортизол и альдостерон — основные и наиболее изученные гормоны коры надпочечников. Результаты исследований, которые носят единичный характер, чаще свидетельствуют об их росте при ХОБЛ [35–38]. При этом в одной из работ при статистически недостоверном повышении уровня кортизола зафиксирована его прямая корреляция с интенсивностью кашля, лейкоцитозом и лейкоцитарным индексом интоксикации и обратная связь с ИМТ [38]. Однако подобное соотношение необъяснимо и пока не является практически ценной информацией, тем более учитывая, что имеются данные о нормальном и пониженном уровнях кортизола у больных ХОБЛ [39]. Что касается минералокортикоидной функции коры надпочечников при ХОБЛ, то есть сведения об увеличении содержания альдостерона в крови больных с данной патологией. Также установлено, что его увеличение коррелирует с нарастанием тяжести заболевания, степенью оксигенации. Рост альдостерона в крови в конечном итоге способствует формированию ЛГ и хронического легочного сердца [40, 41]. Это указывает на то, что в данном случае может иметь место вторичный гиперальдостеронизм, но речь о контринсулярном действии здесь вряд ли может идти.

Определенный интерес с позиции инсулярного/контринсулярного действия могут представлять андрогены, возможные колебания исходного уровня которых в результате могут иметь некоторые гендерные отличия. Так, у мужчин дефицит андрогенов, в частности тестостерона, характеризуется проинсулярным действием, так как он предрасполагает к метаболическому синдрому и СД2. У женщин к нарушениям углеводного обмена могут приводить гиперандрогенные состояния, такие, например, как синдром поликистозных яичников. Они связаны с ИР, непереносимостью глюкозы и последующим развитием СД2 [42]. То есть в этом случае проявляется контринсулярный эффект андрогенов. Интерес к возможным последствиям дефицита андрогенов усилен тем, что за последние годы появились работы, указывающие, с одной стороны, на патогенетическую связь ХОБЛ с низким уровнем тестостерона, возникающим в результате применения глюкокортикоидов при ХОБЛ и под влиянием гипоксии и хронического воспаления. С другой стороны, андрогенный дефицит способствует нарушению механики дыхания за счет уменьшения дыхательной мышечной массы и, соответственно, снижению толерантности к физической нагрузке [43]. Таким образом, усиливается «порочный круг» в патогенезе коморбидности ХОБЛ и СД2. Однако наличие прямой связи андрогенного дефицита, в частности низкого уровня тестостерона, и СД2 неочевидна и требует дополнительного подтверждения. Возможно, некоторые факторы образа жизни, повышающие риск СД2, также повышают риск низкого уровня тестостерона. На это также указывает тот факт, что здоровое питание, физические упражнения и способы лечения оказывают положительный эффект на коррекцию как уровня тестостерона, так и СД2. Ну и, наконец, интерес с позиции вышеизложенного представляет непосредственная положительная роль андрогенов в реабилитации больных ХОБЛ. На основании систематического обзора большого количества работ и метаанализа был сделан вывод о том, что у больных ХОБЛ имело место значимое снижение уровня тестостерона и терапия тестостероном улучшает результаты физической нагрузки, а именно пиковую силу мышц и пиковую нагрузку [44].

За последние годы установлено, что дефицит тестостерона, коррелируя с увеличением ИР, более высокими уровнями триглицеридов и наличием гипертонии, связан с различными компонентами метаболического синдрома. Этот синдром сопряжен с риском развития сердечно-сосудистых заболеваний, являющихся основной причиной летальности при СД2. Заместительная терапия тестостероном у пациентов с дефицитом тестостерона и СД2 и/или метаболическим синдромом показала снижение резистентности к инсулину, общего холестерина, холестерина липопротеинов низкой плотности и триглицеридов и улучшение гликемического контроля [45]. Сравнительная характеристика состояния гормонального фона у курящих и некурящих мужчин со стабильным течением ХОБЛ показала обратную корреляцию показателей уровня тестостерона в крови со степенью тяжести, стажем курения, выраженностью кашля и прямую корреляцию со значениями объема форсированного выдоха за 1-ю секунду [46]. Поэтому изучение механизма положительного действия на гомеостаз инсулина и глюкозы эстрогенов, синтезируемых из тестостерона, является весьма перспективным направлением в плане коррекции нарушений углеводного обмена при ХОБЛ, о чем свидетельствуют работы в этом направлении [46][47].

Имеются сведения о снижении выработки другого андрогена — дегидроэпиандростерона сульфата (ДГЭАС) при ХОБЛ, вырабатываемого в основном корой надпочечников и лишь в 8–10% гонадами. Также отражается взаимосвязь его уровня в крови с клиническими особенностями заболевания, показателями обструкции дыхательных путей, гипоксемией и гиперкапнией, фазой обострения, ремиссией и тяжестью заболевания [47–50]. У больных ХОБЛ установлено понижение уровня ДГЭАС в крови и, наоборот, повышение уровня альдостерона и кортизола. Эти процессы связываются с обострениями и тяжестью заболевания. Также исследователи выяснили, что существует обратная связь содержания ДГЭАС с длительностью заболевания, стадией ХОБЛ, клинической картиной [50].

Примечателен факт снижения ДГЭАС у больных ХОБЛ, выявленный в аспекте исследования биологического старения у них [51]. Он является дополнительным подтверждением того, что, с одной стороны, возраст свыше 60 лет является фактором риска развития СД2, с другой — указывает на влияние уровня КИГ на состояние углеводного обмена. Относительно связи уровня содержания в крови гормонов щитовидной железы и ХОБЛ можно отметить, что в нескольких работах зарегистрирован факт снижения в крови больных ХОБЛ общего трийодтиронина (Т3) [38]. Однако интерпретация этих данных и оценка возможностей их использования встречают у нас затруднения. При этом полученные данные, указывающие на повышенный оксидативный стресс в условиях низкой Т3-ХОБЛ и, вероятно, необходимость в модуляции антиоксидантной системы, возможно, в данном случае заслуживают внимания [52]. Наряду с этим установлена обратная корреляция между уровнем общего Т3 и тяжестью заболевания, степенью гипоксемии и медиаторами провоспаления. Выявлена связь изменения в функции легких и газов артериальной крови с концентрациями свободного трийодтиронина (FT3) у пациентов с ХОБЛ [53]. Вместе с тем тесты функции щитовидной железы у пациентов с ХОБЛ показали значительное увеличение средних значений FТ3 [54]. По мере увеличения тяжести ХОБЛ средние значения свободного Т3 значительно увеличивались. При этом отмечена значительная отрицательная корреляция между уровнями FТ3 и парциальным давлением кислорода (PaO2) и положительная корреляция между уровнями общегоТ3 и парциальным давлением углекислого газа (PaCO2). Значительная отрицательная корреляция наблюдалась между уровнем FТ3 и легочными функциональными тестами пациентов. Продемонстрирована значительная взаимосвязь между уровнем тиреотропных гормонов и частотой обострений ХОБЛ, что предполагает целесообразность диагностики нарушения функции щитовидной железы с целью уменьшения количества обострений и улучшения качества жизни пациентов с ХОБЛ [55].

Таким образом, неразрывная функциональная связь КИГ с инсулином, обеспечивающая гомеостаз глюкозы, и значительная патогенетическая связь ХОБЛ и нарушений углеводного обмена, в которой инициирующую роль играет ХОБЛ, что мы проиллюстрировали в упрощенной схеме (рисунок), предполагает немалую роль КИГ в патогенезе коморбидности ХОБЛ и СД2.

**Figure:**
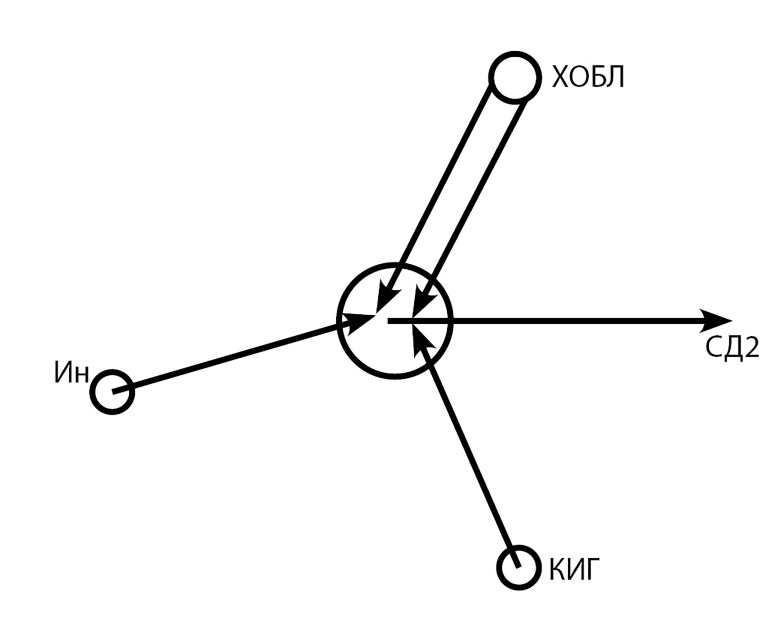
Рис. Звенья гормональной системы, обеспечивающей гомеостаз глюкозы, их взаимоотношения, роль хронической обструктивной болезни легких в их нарушениях и патогенезе сахарного диабета 2 типа.

При этом можно подчеркнуть, что изменение уровня КИГ влияет на одно из ключевых звеньев «порочного круга» в патогенезе данной коморбидности, в частности на оксидативный стресс, усугубляя его. Именно это звено во многом является узловым, сопровождая длительное системное воспаление при ХОБЛ, обусловливая развитие ИР и гипергликемии, на фоне которой повреждаются бета-клетки, и процесс, прогрессируя, способствует развитию СД2 или усугубляет течение уже имеющегося патологического процесса.

## ЗАКЛЮЧЕНИЕ

Анализ литературы свидетельствует, что КИГ играют весьма заметную роль в развитии СД2 при ХОБЛ, воздействуя и усугубляя его ведущие патогенетические звенья, являющиеся во многом общими для этих социально значимых заболеваний. Однако, несмотря на потенциальную важность КИГ в патогенезе СД2 при ХОБЛ, эти проблема остается неизученной. Имеются лишь ограниченные и противоречивые данные об уровне гормонов коры надпочечников, которые пока не представляют возможности их практического применения. Аналогичная картина касается данных по исследованию гормонов щитовидной железы у данного контингента больных. И интерпретация факта снижения в крови больных ХОБЛ Т3 встречает затруднения как в аспекте их интерпретации, так и использования. Некоторое понимание и практический интерес представляют результаты исследования андрогенов при коморбидности ХОБЛ и СД2, указывающие на связь СД2 и изменения уровня тестостерона в этом случае, что открывает определенные диагностические и лечебные перспективы. Но это требует еще уточнения и соответствующих разработок. Поэтому проблема роли КИГ в патогенезе СД2 при ХОБЛ, оставаясь малоизученной, является большим полем для деятельности ее исследователей. Все это не только нацеливает на изучение патогенеза коморбидности ХОБЛ и СД2 и расширение знаний в этом плане, но и составляет перспективы для совершенствования диагностики и терапевтического воздействия в плане коррекции нарушений углеводного обмена при ХОБЛ.
